# Why is it important to implement meta-research in universities and institutes with medical research activities?

**DOI:** 10.3389/frma.2025.1497280

**Published:** 2025-03-19

**Authors:** Ivan David Lozada-Martinez, Dionicio Neira-Rodado, Darly Martinez-Guevara, Hary Salome Cruz-Soto, Maria Paula Sanchez-Echeverry, Yamil Liscano

**Affiliations:** ^1^Biomedical Scientometrics and Evidence-Based Research Unit, Department of Health Sciences, Universidad de la Costa, Barranquilla, Colombia; ^2^Universidad de la Costa, Barranquilla, Colombia; ^3^Grupo de Investigación en Salud Integral (GISI), Departamento Facultad de Salud, Universidad Santiago de Cali, Cali, Colombia

**Keywords:** meta-research, biomedical research, needs assessment, evidence gaps, publications, knowledge discovery

## Abstract

In recent years, there has been a growing concern over questionable practices and a lack of rigor in scientific activities, particularly in health and medical sciences. Universities and research institutes are key players in the development of science, technology, and innovation. Academic institutions, whose primary mission is to generate and disseminate knowledge, bear the responsibility in many parts of the world to act as consultants and guardians of scientific integrity in health research. Then, universities and research institutes must act as guardians of the research and technological development process, utilizing methodological and operational evaluation tools to validate the rigor and quality of medical research. Meta-research is defined as the research of research itself. Some of the most important specific objectives of meta-research include the assessment of research relevance, the evaluation of evidence validity, and the exploration of scientific integrity. A significant portion of evidence in the medical and health sciences literature has been found to be redundant, misleading, or inconsistent. Although this issue is of great importance in global health, discussions about practical and tangible solutions remain fragmented and limited. The aim of this manuscript is to highlight the significance of employing meta-research within universities and research institutes as a tool to monitor scientific rigor and promote responsible practices in medical research.

## 1 Introduction

In recent years, there has been a growing concern over questionable practices and a lack of rigor in scientific activities, particularly in health and medical sciences (Mayta-Tristán et al., 2024; Phogat et al., 2023). These instances of scientific misconduct have profound repercussions, often overlooked by the general population (National Academies of Sciences Engineering Medicine; Policy Global Affairs; Committee on Science, 2017), but increasingly highlighted through scientific literacy and the broader dissemination of science. In response, various researchers, groups, and institutions have developed strategies and metrics aimed at critically and objectively evaluating scientific integrity (Munaf et al., 2017; Chalmers et al., 2014). While this is often measured by publication frequency, there are deeper epistemological dimensions and interpretations, as well as implications for medical practice and social responsibility (Munaf et al., 2017; Chalmers et al., 2014). This is particularly relevant to the intersection of knowledge generation, its transfer, and the influence that health decision-making based on this knowledge has on society (Rahman and Ankier, 2020). The protection of scientific integrity and the generation of new knowledge in health are intrinsically linked to the safeguarding of human rights, human security, and public health outcomes (Khadilkar, 2018).

Universities and research institutes are key players in the development of science, technology, and innovation (Geng and Yan, 2021). In the medical and health sciences, these institutions frequently collaborate with university hospitals, clinical trial centers, and clinical research centers, which provide access to primary data, financial resources, advanced equipment, specialized professionals, and opportunities for international collaboration (Institute of Medicine (US), 2002). This synergy enables the development of increasingly high-quality medical research. Academic institutions, whose primary mission is to generate and disseminate knowledge, bear the responsibility in many parts of the world to act as consultants and guardians of scientific integrity in health research (Robishaw et al., 2020). They are tasked with ensuring the quality of knowledge that informs medical practice. Thus, it is academia itself, primarily represented by universities and research institutes, that must deploy the necessary tools to address lapses in scientific rigor within medical research.

Although scientific rigor and quality research is of great importance in global health, discussions about practical and tangible solutions remain fragmented and limited. The aim of this manuscript is to highlight the significance of employing meta-research within universities and research institutes as a tool to monitor scientific rigor and promote responsible practices in medical research.

## 2 Growth of global scientific production in medical sciences over time

In recent decades, there has been a phenomenon of rapid expansion of global scientific output. The outbreak of coronavirus disease 2019 (COVID-19) pandemic further intensified this trend, with some countries increasing their annual medical and health sciences publication output by more than 40% in recent years (Oliveira et al., 2022; Zhang, 2021). This astounding increase brought simmering concerns over publication volume to the forefront and suggested a potential crisis in the field, in addition to general concerns over publication quality (Abbott et al., 2022). The sheer volume of clinical evidence became so vast that it was impossible to rigorously and promptly analyze the data, leading to significant uncertainty in health-related decision-making (Pan American Health Organization, 2024).

This increase sparked a discussion about the excessive number of opinion pieces, commentaries, perspectives, and editorials (Lozada-Martínez et al., 2021). Concerns arose over potential conflicts of interest and the personal benefits of rapidly publishing in indexed journals, even when the resulting literature offered little to no meaningful contribution to the academic community (Lozada-Martínez et al., 2021; Federico, 2020). In response to these concerns, authors have justified the rate of publication as needing to address the 'publish or perish' phenomenon, which reflects the tension between the quantity and quality of publications (Suart et al., 2022). This issue of overpublishing low quality and low impact studies becomes more pronounced when publications are evaluated based not on the quality and potential real-world impact of the scientific evidence itself, but rather on journal metrics like publication rate, impact factor, and prestige (CITE). Consequently, the excessive number of short papers may be associated with the 'publish or perish' phenomenon (Laine et al., 2025).

With the updating of metrics in citation indexes and databases, such as the Scimago Journal and Country Rank (SJR), it became evident that the volume of publications from certain countries had increased by more than 40% compared to just a few years earlier, a trend that warrants closer examination (Lozada-Martinez et al., 2022). While publication frequency alone is not a metric of quality, it is essential to carefully assess the characteristics of scientific output—such as the types of manuscripts, affiliations, scientific and technological capacities, collaboration, and author networks—to ensure that the growth in scientific production aligns with the capabilities of the institutions and research groups. Otherwise, it raises red flags regarding potential research and publication misconduct, particularly when there is no valid justification for the origin of the data, time of analysis and interpretation, or attribution of authorship (Mayta-Tristán and Borja-García, 2022).

In this context, universities and research institutes must act as guardians of the research and technological development process, utilizing methodological and operational evaluation tools to validate the rigor and quality of the research. In recent years, there has been a significant increase in cases of questionable conduct in medical research, including an unjustified rise in scientific production (Baumeister et al., 2021; McDermott et al., 2024; Jung et al., 2021). This trend has raised concerns about potential data falsification, low-quality research, and “salami” publications. Notably, this phenomenon became particularly evident during the pandemic and post-pandemic phases (Baumeister et al., 2021; McDermott et al., 2024; Jung et al., 2021; Mayta-Tristan, 2024). Thus, while global scientific and technological progress is directly correlated with nations' ability to generate new knowledge (Munaf et al., 2017; Chalmers et al., 2014), it is evident that the abrupt and rapid growth of scientific production in certain countries presents significant challenges regarding scientific relevance and pertinence that must be addressed (Munaf et al., 2017; Chalmers et al., 2014).

## 3 Uncertainty and questionable practices in medical research and scientific publication

Although numerous examples and scenarios related to questionable or uncertain practices in medical research have historically existed, raising concerns about the validity of evidence, the recent surge of publications generated during the COVID-19 pandemic represents a notable academic case for analysis (Schonhaut et al., 2022). This phenomenon posed a significant challenge in editorial management and meta-research, as suspicious findings emerged regarding the quality of some representative studies.

Due to the need to accelerate research processes, a variety of study designs were developed and executed to understand the pathophysiology, complications, and potential therapies for COVID-19, including its variants and disease phenotypes (Sousa Neto et al., 2023). Unfortunately, with the emergence of numerous clinical trials and systematic reviews, there was significant uncertainty regarding the quality of clinical evidence and evidence-based recommendations (Baumeister et al., 2021; McDermott et al., 2024; Jung et al., 2021). Meta-epidemiological studies demonstrated that original COVID-19 research, when compared to historical studies, had a significantly shorter median acceptance time (13 vs. 110 days; *p* < 0.001; Jung et al., 2021). Regardless of the study design used, all COVID-19 studies had significantly lower median methodological quality scores (as assessed by the Newcastle–Ottawa Scale and QUADAS-2 for observational and diagnostic studies, respectively) compared to historical studies. Diagnostic studies on COVID-19 exhibited the highest risk of bias (93.6%; Jung et al., 2021). However, there were notable shortcomings in the fulfillment of essential methodological quality criteria in randomized controlled trials, such as sequence generation risk of bias, allocation concealment, blinding of participants and personnel to all outcomes, blinding of outcome assessors for all outcomes, and selective outcome reporting (Jung et al., 2021). These findings led to the conclusion that the low quality of evidence found in some COVID-19 studies generated substantial uncertainty, particularly regarding the certainty of the evidence and the methodological quality of the studies.

Other exploratory and comparative analyses identified a similar trend when evaluating the methodological and reporting quality of systematic reviews that served as the basis for health decision-making during the COVID-19 pandemic (Baumeister et al., 2021; McDermott et al., 2024). Therefore, one of the most significant criticisms was the implication of using evidence of very low quality or uncertain certainty for mass health decision-making during the global health crisis.

This uncertainty further increased during the transition to the post-pandemic phase, as concerns about the quality and rigor of evidence were accompanied by a notable number of retractions of original COVID-19 studies due to questionable research practices and deficiencies in the peer review process (Schonhaut et al., 2022; Taros et al., 2023). The acceleration of the review and acceptance processes in journals (on average, < 10 days), as well as ethical and scientific concerns (Schonhaut et al., 2022), highlighted the lack of control over the quality of medical research—not only by journals but also by institutions. It became apparent that there were specific niches where a common group of authors had numerous retractions (Schonhaut et al., 2022; Taros et al., 2023).

Interestingly, a scientometrics analysis revealed that even after the retraction of these documents, they continued to be cited up to 45 [standard deviation (SD) 138.9] times more than the average article in the Scopus database (*p* = 0.01; Taros et al., 2023). In 3 out of 10 retracted articles (*n* = 27/90), the guidelines established by the Committee on Publication Ethics (COPE) were not followed, preventing the identification of the reasons for the retraction (Taros et al., 2023). Although the pandemic and post-pandemic phase created an intense scenario in which this phenomenon may have been exacerbated, researchers in the field of meta-research generally emphasized the importance of transparency and clearly understanding the direct causes of withdrawals and retractions, given the implications for health policies, public health, and medical interventions that arise from the use of data of questionable quality (Besançon et al., 2021; Stoto et al., 2022; Raynaud et al., 2021; Lozada-Martinez et al., 2024).

The lessons learned from research processes during the COVID-19 pandemic demonstrated that open science, and the careful enforcement of scientific rigor and transparency in medical research, saves lives (Besançon et al., 2021). Misinformation, infodemics, and questionable research practices create confusion within the general community, academia, and healthcare workers, disrupting the consistency and coherence needed to implement effective health prevention and education strategies. Therefore, the responsibility to monitor and correct questionable practices and uncertainty in medical research lies with society as a whole, but especially with those equipped with technical and methodological tools (academia and the state).

## 4 Meta-research in medical sciences: a tool to reduce the scientific fracture and monitor scientific activity

Meta-research is defined as the research of research itself (Ioannidis, 2018; Ioannidis et al., 2015; Ioannidis, 2016). Given that science and innovation are key to human progress and the generation of health knowledge that advances human security, it is essential to ensure the highest standards of research in medical sciences to achieve a real impact on the population (Ioannidis, 2018). Meta-research encompasses five domains of focus: methods, reporting, evaluation, reproducibility, and incentives in science (Ioannidis et al., 2015; Ioannidis, 2016). Some of the most important specific objectives of meta-research include the assessment of research relevance, the evaluation of evidence validity, and the exploration of scientific integrity (Ioannidis, 2016). This emerging discipline, therefore, has the potential to address the challenges and opportunities in studying and strengthening scientific activity.

Medical and health sciences are arguably the fields where meta-research has been most extensively applied in recent years (Rahman and Ankier, 2020). Over the past two decades, the increasing volume of scientific publications in medical sciences made it necessary to develop tools to assess the true value and practical utility of clinical evidence (Rahman and Ankier, 2020; Robishaw et al., 2020). Similarly, it has become essential to employ methods capable of identifying evidence that presents serious concerns and poses risks to the community (Mayta-Tristán et al., 2024).

Through meta-research, it has been identified that some systematic reviews and meta-analyses in medical and health sciences have been redundant, misleading (by overestimating or underestimating the effects of interventions), and inconsistent (Lund et al., 2022). Initially, it has been observed that the growth of systematic review publications has reached “epidemic proportions” (Lund et al., 2022). In some areas of medical research, such as the use of antidepressants, the frequency of overlapping studies is particularly high. Moreover, significant conflicts of interest associated with the industry have been found in these publications (Lund et al., 2022).

Given the relevance and importance of medical research, meta-research in health sciences has revealed opportunities to address knowledge gaps and align them with the real needs of society (Luchini et al., 2021). This does not only pertain to the various knowledge areas and disciplines but also to the methodological limitations of previous studies that must be addressed to improve the quality and certainty of evidence used for decision-making in health (Luchini et al., 2021). These gaps should be evaluated from the characteristics of the study design protocol to the perceptions of the end-users of the information, who are theoretically the ones to benefit from this new knowledge (Luchini et al., 2021). To achieve this goal, there are guidelines and tools available to assess domains of interest and uncover the real and priority gaps (Luchini et al., 2021). Previous research has shown that the use of these guidelines allows researchers to specifically identify which items are not fulfilled during the design or reporting of clinical studies, affecting reproducibility, reliability, and confidence in the evidence within health sciences (Grosman and Scott, 2022).

Over the years, with the adoption of new guidelines that strengthen the reporting and methods of clinical studies, there has been a notable improvement in the quality patterns of clinical research (Nguyen et al., 2022). Additionally, the continuous evaluation of scientific practice has helped identify preventable and overlooked errors throughout history, even among Nobel laureates (Else, 2024) or in cases of scientific fraud (Orfila, 2023), which unfortunately is a phenomenon that is becoming increasingly frequent and threatens the rigor and integrity of science. Therefore, promoting meta-research as a tool to reduce the scientific fracture and monitor scientific activity should be a responsibility of key stakeholders in community-oriented medical research, including academia (represented by universities and institutes) and the government.

## 5 The role of institutions in meta-research: strengthening scientific integrity and research accountability

Academic institutions, particularly universities and research institutes, play a critical role in shaping the scientific landscape by generating, disseminating, and applying knowledge in health and medical research (Ioannidis, 2018; Ioannidis et al., 2015). Beyond their role as knowledge producers, these institutions must function as scientific auditors, ensuring that research practices align with methodological rigor, transparency, and ethical principles (Macleod et al., 2014; Ioannidis, 2014; Begley and Ioannidis, 2015). The increasing volume of scientific publications, combined with concerns regarding low-quality research, necessitates institutional engagement in meta-research to uphold the credibility of scientific outputs and reinforce the social responsibility of academia (Ioannidis, 2018; Ioannidis et al., 2015; Ioannidis, 2014; Begley and Ioannidis, 2015).

Meta-research, defined as the study of research itself, provides universities and research institutes with an essential mechanism to evaluate and improve the quality of scientific publications (Ioannidis, 2018). While regulatory bodies and funding agencies establish broad guidelines for research integrity, institutions are uniquely positioned to implement proactive, data-driven approaches that scrutinize the reliability, reproducibility, and validity of medical research. Universities and research institutes possess inherent advantages that make them suitable for this role (Macleod et al., 2014; Ioannidis, 2014), including:

- ***Access to research ecosystems***: Universities and institutes operate within structured research environments that facilitate direct oversight of ongoing projects. Research ethics committees, institutional review boards (IRBs), and data monitoring committees can be expanded to incorporate meta-research principles, systematically assessing methodological soundness and reporting accuracy in published and ongoing studies.
- ***Expertise in methodological and statistical rigor:*** Academic institutions house experts in epidemiology, biostatistics, and methodology who are capable of critically appraising research outputs. By institutionalizing meta-research units, universities can standardize best practices for evaluating study designs, ensuring appropriate statistical analyses, and detecting biases that compromise research validity.
- ***Capacity for cross-disciplinary collaboration:*** The inherently interdisciplinary nature of universities fosters collaboration between medical researchers, data scientists, ethicists, and policy experts. This integration facilitates comprehensive assessments of research quality, enabling institutions to address systemic issues such as publication bias, selective reporting, and research waste.
- ***Institutional autonomy and academic freedom***: Unlike external regulatory bodies, universities and research institutes maintain a degree of autonomy that allows for independent and unbiased evaluations of scientific integrity. This independence strengthens their ability to implement rigorous quality control mechanisms without external political or financial pressures influencing their decisions.
- ***Influence on research culture and training***: As primary centers of scientific training, universities shape the research habits of emerging scientists. Embedding meta-research principles in postgraduate curricula, doctoral training programs, and faculty development initiatives ensures that future researchers adopt high standards of rigor and transparency from the outset of their careers.

Why meta-research should be an institutional priority? Despite growing awareness of research misconduct and questionable publication practices, many institutions remain reactive rather than proactive in addressing these challenges (Ioannidis, 2014; Begley and Ioannidis, 2015). The assumption that scientific journals and peer review systems serve as sufficient safeguards against low-quality research is demonstrably flawed, as evidenced by the high prevalence of retracted publications and reproducibility crises across medical sciences (Ioannidis et al., 2015; Ioannidis, 2016). Institutions must assume a more direct role in addressing these deficiencies by integrating meta-research into their research governance frameworks.

Institutions that actively engage in meta-research can enhance their academic reputation and credibility by demonstrating a commitment to high-quality, evidence-based inquiry. This approach not only benefits individual researchers but also strengthens the institution's competitiveness in securing grants, forming international collaborations, and influencing health policies based on robust scientific evidence (Ioannidis, 2018; Ioannidis et al., 2015; Ioannidis, 2016; Lund et al., 2022).

Furthermore, meta-research aligns with the mission of universities to serve as knowledge custodians for society. By scrutinizing the validity of published medical research, institutions can prevent the dissemination of misleading findings that could negatively impact clinical practice, public health policies, and patient outcomes (Ioannidis, 2018).

**Figure 1:**
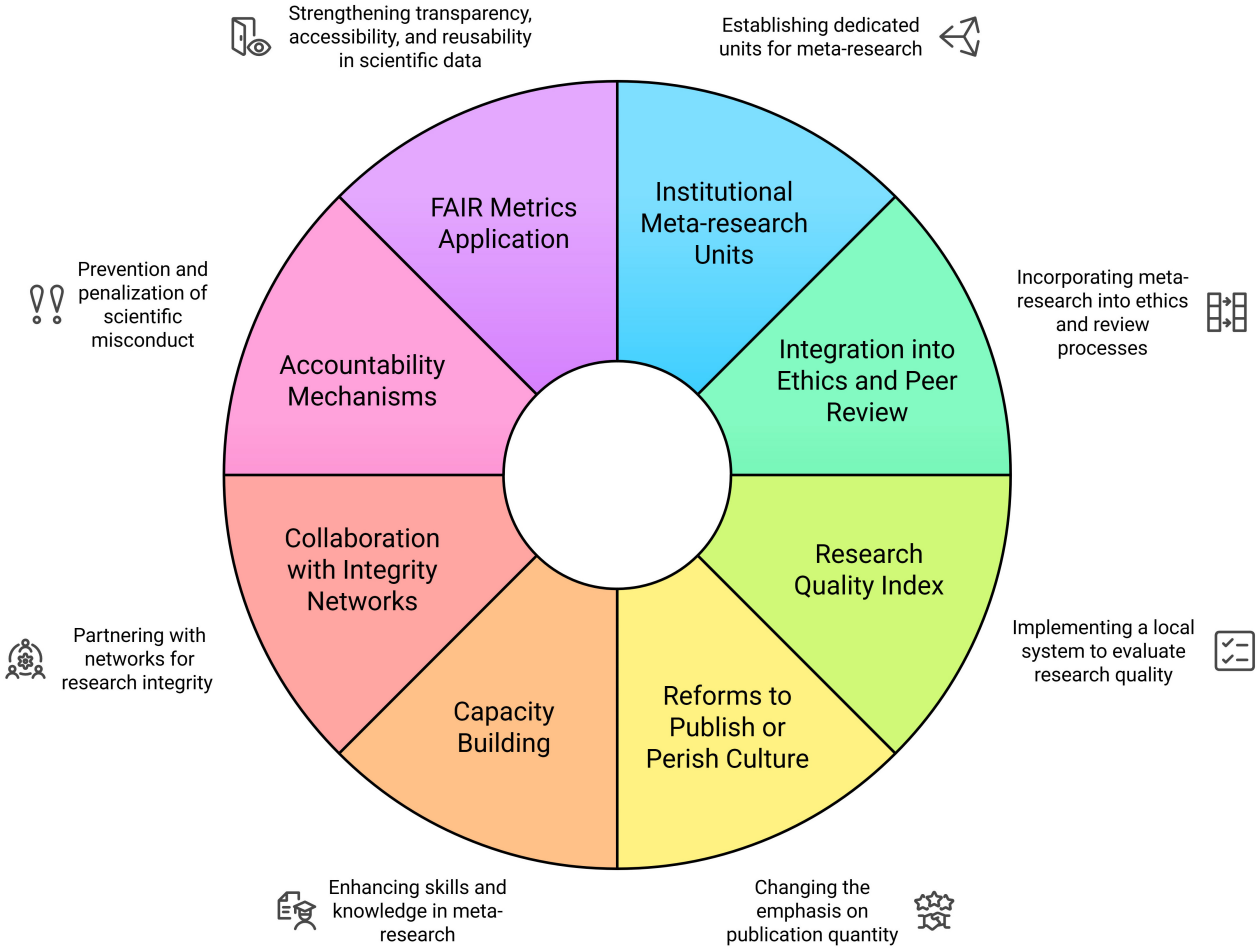
A proposed institutional framework for meta-research implementation in academic institutions. Source: authors.

While there is limited published evidence regarding the effectiveness of specific institution-level interventions to enhance the quality of publication practices, there are a number of potential opportunity areas to integrate meta-research and quality initiatives into existing institutional structures and functions. Establishing institutional policies and local meta-research efforts to evaluate and address factors influencing the researcher experiences that drive research behaviors, could serve as a means to engage with the need for improved quality regulation in academic publishing and research practices. We propose the following intervention points that may serve as opportunities to operationalize meta-research in academic institutions (Figure 1):

1. ***Establishment of institutional meta-research units:*** Dedicated meta-research units should be created within faculties of medicine, public health, and biomedical sciences, staffed by methodologists, statisticians, and experts in scientific integrity. Their primary role would be to systematically evaluate institutional research output and detect questionable research practices.
2. ***Integration of meta-research into research ethics and peer review***: Institutional review boards should incorporate meta-research methodologies to assess study protocols before approval. Internal peer review mechanisms should be strengthened, and post-publication audits should be conducted to ensure research transparency and impact.
3. ***Implementation of a research quality index:*** A standardized institutional research quality index should be developed to assess study design, transparency, reproducibility, and ethical compliance, providing measurable indicators of research integrity and institutional performance. These indicators must be adapted to the institution's local health, social, economic, political and cultural context. Quality indices for research have previously been proposed and replicated in other fields, yielding interesting and useful results worth considering (Sharma, 2012; Zeraatkar et al., 2017; Pluskiewicz et al., 2019).
4. ***Reforms to the “publish or perish” culture***: Traditional evaluation metrics (e.g., impact factor, citation counts) should be replaced with quality-focused frameworks that prioritize methodological rigor. Researcher performance should be assessed based on transparency and reproducibility, with incentives for faculty engaged in high-quality peer review, open science, and data-sharing initiatives.
5. ***Capacity building in meta-research***: Formal training programs in meta-research should be introduced for faculty, postgraduate students, and early-career researchers, covering bias detection, methodological quality assessment, and systematic error identification. Collaborative networks should be established to share best practices.
6. ***Collaboration with national and international research integrity networks:*** Universities should participate in global research integrity initiatives, contributing data and expertise to enhance scientific standards. Engagement in meta-research consortia would facilitate knowledge exchange and the development of standardized protocols for assessing research quality.
7. ***Integration of Accountability Mechanisms and Sanctions:*** One of the most significant challenges for the effective implementation of meta-research in universities and research institutes engaged in medical research is the lack of explicit mechanisms to ensure accountability and enforce sanctions for breaches of scientific integrity. Various authors have noted that the absence of a robust coercive framework can undermine efforts to improve research quality and reduce misconduct (Craig and Taswell, 2018; Taswell, 2025; Craig and Taswell, 2024; Taswell, 2024). It is argued that meta-research, when embedded within an institutional ecosystem that reinforces scientific integrity through targeted governance strategies, may contribute to a structural change in the monitoring and assurance of research quality (Craig and Taswell, 2018).
  Accountability in scientific production should not depend solely on the oversight of academic journals or external regulatory agencies (Taswell, 2024); rather, it must be incorporated into the structure of universities and research centers through specific policies designed to prevent and penalize misconduct (Taswell, 2024). The imposition of sanctions within universities and research institutes should be proportional to the severity of the violation (Craig et al., 2022). While methodological errors can be rectified through review processes, intentional scientific fraud should result in more severe measures, including the prohibition of receiving funding or the disqualification from continuing research activities.
  To prevent the implementation of meta-research from being perceived as an abstract ideal, it is essential that institutions adopt clear and functional strategies that translate findings into concrete actions. Active monitoring of scientific practices, combined with the imposition of tangible consequences for proven cases of fraud or negligence, may foster an environment of increased responsibility and trust in scientific production (Taswell, 2024; Craig et al., 2022).
8. ***Application of the FAIR (Attribution to Indexed Reports) Family of*** ***Metrics in Evaluating the Accessibility, Interoperability, and Reusability*** ***of Scientific Data***: The integration of FAIR metrics within institutional evaluation models presents a key opportunity to enhance transparency, accessibility, and reusability in scientific data (Craig and Taswell, 2018). The FAIR concept has been proposed as a normative framework to improve research quality and reproducibility by establishing principles that facilitate the independent verification of scientific results and the traceability of data used in biomedical studies (Craig and Taswell, 2018; Taswell, 2025; Craig and Taswell, 2024; Taswell, 2024).

This framework consists of five metrics: two assess adherence to best practices—the number of correctly attributed background statements and the number of genuinely original claims. The remaining three identify deviations from best practices—the number of misattributed background statements, the number of background statements lacking proper references, and the number of claims falsely presented as original (Craig and Taswell, 2018). By guiding researchers in properly documenting the accessibility and reusability of their data, FAIR metrics can reduce the incidence of scientific fraud and enhance the reliability of meta-analyses and systematic reviews, which depend on the availability of primary data for accurate execution (Craig et al., 2023, 2019).

The evolving challenges of scientific integrity necessitate that universities and research institutes embrace meta-research as a fundamental institutional responsibility. Rather than functioning solely as producers of research, academic institutions must adopt a dual role as evaluators of knowledge quality, ensuring that medical research serves its intended purpose—advancing human health and wellbeing. By implementing structured meta-research programs, institutions can lead the transformation toward a more transparent, reliable, and impactful scientific ecosystem.

## 6 Conclusions

This perspective emphasizes the importance of utilizing meta-research within universities and research institutes as a key tool for monitoring scientific rigor and promoting responsible practices in medical research. By integrating meta-research into institutional frameworks, developing clear guidelines, fostering interdisciplinary collaboration, and enhancing transparency, these institutions can effectively safeguard the quality and integrity of scientific output. Additionally, establishing educational pro-grams in meta-research will further equip researchers with the necessary skills to ensure high standards in medical research and its application to public health.

## Data Availability

The datasets presented in this article are not readily available because none. Requests to access the datasets should be directed to Ivan David Lozada-Martinez, ilozada@cuc.edu.co.
